# Heightened emotion processing as a compensatory mechanism in persons with Alzheimer's disease: Psychological insights from the tri-network model

**DOI:** 10.3389/frdem.2022.983331

**Published:** 2022-09-30

**Authors:** Alison Warren

**Affiliations:** The Department of Clinical Research and Leadership, George Washington University School of Medicine and Health Sciences, Washington, DC, United States

**Keywords:** Alzheimer's disease, dementia, emotion, social, communication, network

## Abstract

Social and emotional communication is an integral tenant of life quality and well-being. Aberrations in functional connectivity can alter social emotional behavior in numerous disease states, including dementia. This paper aims to review the major network changes observed in Alzheimer's disease, with a focus on the tri-network model. The central executive network, default mode network, and principally the salience network will be discussed as they relate to both pathology and compensatory behavioral manifestations in persons with dementia. The psychological and behavioral correlates of these network changes will be reviewed with the intent of increasing understanding about the conscious experience and communication modalities utilized by persons with dementia, the understanding of which may promote meaningful communication with care providers and loved ones. This paper further seeks to reframe social emotional communication methods used by persons with dementia by marrying current knowledge of neuroscience, psychology, and person-centered care. In this way, a perspective is offered that considers the heightened emotional states experienced by persons with dementia as a potential compensatory mechanism that may hold practical value under some circumstances. The many ways in which the brain adapts to physical and psychological changes, aging, and injury are still under exploration. Emotion processing may provide clinical insight into the subjective experience of dementia in this regard. Emotions, therefore, may serve to promote social bonds, provide an avenue for non-verbal communication, and act as a construct to maintain agency in persons who ultimately lose autonomy.

## Introduction

As the most common form of dementia, Alzheimer's disease (AD) represents a major global health crisis, affecting over 50 million people worldwide and expected to double every 20 years (Grande et al., 2020). The enormous magnitude of people affected by AD, including their families and caregivers, underscores the great challenge at hand, along with the major financial burden it poses on individual and societal levels, estimated at $818 billion in 2015 (Hallschmid, 2021). Despite its prevalence and high level of detriment, there are no causal treatments nor cures (Hallschmid, 2021). AD is characterized by beta-amyloid (Aβ) plaque deposition and neurofibrillary tangles (Sturm et al., 2013) that result in progressive neurocognitive degeneration (Liu et al., 2018), typically beginning in the medial temporal lobe (Sturm et al., 2013; Olajide et al., 2021).

Memory impairment is often accompanied by a variety of neuropsychiatric symptoms, but with preservation of the full range of human emotion during a substantial duration of the disease course (Lazar et al., 2017). Studies comparing AD to other types of dementia have been consistent with clinical observations, in that, patients with AD retain the ability to recognize emotions and manage successful social interactions (Goodkind et al., 2015). Goodkind et al. (2015) evaluated 24 patients with frontotemporal dementia (FTD) and 23 patients with AD using short film clips depicting 3 emotion categories (positive, negative, and self-conscious). They found emotion recognition in patients with AD was spared across all 3 categories, whereas patients with FTD demonstrated significant deficits (Goodkind et al., 2015). Heightened emotions and emotional changes are also common in the prodromal stage of mild cognitive impairment (MCI), and may even portend an underlying AD process (Sturm et al., 2013). Despite worsening episodic memory performance (Sturm et al., 2013), persons with AD do maintain implicit memory (Sabat, 2006), a heightened sense of social sensitivity (Fredericks et al., 2018), and forms of social contagion, including empathy (Choi and Jeong, 2017).

Several decades of research have been dedicated to uncovering the etiology, pathology, diagnostic markers, and potential treatments for AD. An equally important priority must also address the multitude of persons suffering *currently* to recognize their subjective experience, the understanding of which lends the opportunity to enhance quality of life in persons with dementia and their caregivers. Owing to continually refined imaging methods, the intersection of psychology and neuroscience is beginning to unravel the multifactorial puzzle of the dynamic interplay between brain networks, emotion processing, and the psychological experience of persons with dementia, both objectively and subjectively.

The aim of this narrative review is to provide an overview of the current evidence of functional connectivity as it relates to the social emotional experience, including associated communication processes of persons with dementia. In doing so, it is intended to explore how and to what extent these neuroscientific findings can translate into clinical care, public knowledge, and social interactions that promote personhood. It is further aimed to provide a positive perspective of these compensatory shifts within the brain as a potential protective factor in persons with dementia in the hopes of augmenting the brain-behavior relationship, with a psychological perspective anchored in personhood. To the extent that some of these alterations are predictable, but not always pathological, in patients with AD, a concerted public policy effort can be made to better prepare caregivers to navigate eventual behavioral and emotional changes. Along this premise, this paper will highlight some emotion management techniques observed in persons with dementia, review basic neurobiological pathology of AD and the disruptions observed in the triple network, and explore the psychological and behavioral implications of the salience network. This paper will conclude with potential clinical implications of incorporating this knowledge into the social context of persons with dementia, as well as identification of social-behavioral changes as potential early indicators of cognitive decline.

## Emotional relevance and importance in persons with dementia

Emotions, along with their overt behavioral manifestations, serve as a form of communication across several species (Semin et al., 2019). Moreover, emotion contagion is observed within and between species (i.e., humans to humans, humans to dogs, etc), and often escapes consciousness, which carries important implications for inducing affective states (Semin et al., 2019). This automatic transmission of emotional content between beings is a reflex-like reaction that likely has an evolutionary origin (Sturm et al., 2013; Celeghin et al., 2020). While non-verbal communications derived from emotions can be quite efficacious, they may be easily overlooked in persons with dementia. In fact, non-verbal communications, including emotions, can impart knowledge to caregivers when semantics fail, including unmet needs, which may be misinterpreted as neuropsychiatric symptoms (Cunningham et al., 2019).

We as humans utilize a myriad of techniques to regulate our emotional states. The additional challenges of deteriorating verbal and cognitive abilities inherent in AD may create frustration that catalyzes emotional expression. Despite these barriers, persons with dementia remain creative and effortful in adaptation of communicative behavior, or purposeful lack thereof, such as instances in which they are aware of their deficits and thus avoid speaking due to fear of embarrassment. Further illustrations of this explicit emotion management are the common use of communicative coping behaviors (CCBs). Persons with dementia often utilize CCBs to manage the difficulties of the disease (Saunders et al., 2016), including their internal emotional state. Examples of CCBs include using humor and expressing thankfulness (Saunders et al., 2016). Per this observation, Saunders et al. (2016) created the CCB checklist, which includes behaviors generally categorized as either emotion-focused or activity-focused, that may serve to improve the quality-of-life persons with dementia. In this way, we observe persons with dementia utilizing emotional processing to navigate their cognitive challenges, especially in those who have conscious awareness of their deficits. In those who do not have conscious awareness of their deficits, (known as anosognosia), implicit emotion processing seems to be heightened while explicit control may be dampened. Whether this is also a reflection of network changes that mirror compensatory behaviors, rather than solely a consequence of neurodegeneration, merits exploration.

Clinical studies have revealed that several brain areas that contribute to emotion processing, such as the amygdala, anterior insula, basal ganglia, and frontal cortex (Novak et al., 2012; Mattavelli et al., 2019, 2021; Celeghin et al., 2020), are also targets of many neurocognitive diseases. Neurodegenerative disorders in turn affect emotion processing in a variety of ways. While several brain areas are involved in the manifestation of emotional changes, so too are they are reflection of functional connectivity changes in brain networks. The increase or decrease in network connectivity produces clinical consequences in distinct patterns (Zhou and Seeley, 2014), with associated psycho-emotional manifestations. For example, persons with Parkinson's disease and Huntington's disease often demonstrate a deficit in emotion recognition, associated with degeneration of the basal ganglia and insula (Celeghin et al., 2020; Mattavelli et al., 2021); loss of empathy and interpersonal warmth are seen in frontotemporal dementia (Toller et al., 2019), consistent with salience network atrophy (Toller et al., 2019; Pasquini et al., 2020); and heightened emotions and forms of social contagion are seen in persons with Alzheimer's disease (Choi and Jeong, 2017), relating to medial temporal lobe degeneration (Sturm et al., 2013), decreased default mode network activity, and salience network upregulation (Zhou and Seeley, 2014). Recognizing that each neurodegenerative disease is marked by stereotyped patterns of cellular and brain network changes (Pasquini et al., 2020), this narrative will focus on AD presentations. The poignant study by Zhou and Seeley (2014) remarkably illustrated that specific network alterations can serve as a platform for understanding a wide range of neuropsychiatric alterations in patients (Geschwind and Nestler, 2014; Zhou and Seeley, 2014). Perhaps, these network changes at times exist outside the realm of pathology, and can serve a practical purpose in everyday interactions.

## Brain network disruption in AD

The network disruption in AD is robustly supported to correlate with cognitive deficits and heightened emotions (Badhwar et al., 2017). More specifically, decreased functional connectivity in the central executive network and default mode network, and increased functional connectivity in the salience network respectively (Zhou and Seeley, 2014; Liao et al., 2018; Sarli et al., 2021). Reflection upon these patterns of network disruption may suggest a possibility that the emotions and behaviors that follow may reflect a neuropsychological compensation to preserve a means of communication.

The complex network of structural connections in the human nervous system, known as the ‘connectome’ enables proper functional neural communication and integration (Van den Heuvel and Sporns, 2019). Brain networks include a collection of nodes (brain regions) and edges (connections), often measured with functional MRI (fMRI) to determine functional connectivity, and diffusion tensor imaging to reveal structural connectivity (Menon, 2011). Functional networks are clusters of subregions that operate in a functionally correlated, synchronous manner during cognitive and behavioral tasks (Shaw et al., 2021). Within this large complex functional network, a core triple network model was described by Menon in 2011 encompassing the default mode network (DMN), the salience network (SN), and central executive network (CEN); the dysconnectivity of which is associated with numerous neurocognitive and psychological disorders (Menon, 2011). Generally speaking, the DMN and CEN are functionally antagonistic, and are modulated by the SN that initiates task-switching based on relevant stimuli (Chand et al., 2017; Zhang et al., 2022).

The CEN, anchored in the dorsolateral prefrontal cortex and lateral parietal cortex, is involved in externally directed cognitive tasks such as executive functions, working memory, problem-solving, and decision-making (Liao et al., 2018; Shaw et al., 2021). Coined and confirmed by PET by Raichle et al. (2001) the DMN spans medial and parietal regions, medial prefrontal cortex, hippocampus (Scherr et al., 2021) posterior cingulate cortex (Raichle et al., 2001; Greicius et al., 2004), and precuneus (Raichle et al., 2001). It is considered a resting-state network, involved in internally directed cognitive behaviors such as daydreaming, mind-wandering, focused thought (Raichle, 2015; Shaw et al., 2021), and episodic memory (Greicius et al., 2004). Finally, the SN, anchored mainly in the anterior cingulate cortex and anterior insula (Choi and Jeong, 2017; Snyder et al., 2021), participates in attentional capture of biologically and cognitively related stimuli and events, affective processing (Snyder et al., 2021), as well as subsequent engagement of executive functions (Menon, 2011). The SN modulates the internal and external task switching between the CEN and DMN, which are anticorrelated under the control of the SN, allowing for healthy mentation and emotional management (Shaw et al., 2021). Owing to the dynamic interplay between the three core networks, a disruption in one network tends to impact the other two that manifest as clinical signs and symptoms beyond the primary deficit (Zhang et al., 2022).

Numerous resting state fMRI studies have established AD as a type of “disconnection syndrome” of aberrant functional connectivity, which can serve as a biomarker for AD (Liao et al., 2018). Alterations in the DMN and SN have been observed, including during the early stages (Pereira et al., 2021; Sarli et al., 2021). The DMN exhibits decreased within-network and between-network connectivity (Mondragón et al., 2019) and has been well-described in both early and late onset AD (Schultz et al., 2020). Imaging of AD patients at various stages has demonstrated hypometabolism in the posterior cingulate cortex, right angular gyrus, and inferior parietal lobes, along with disconnection in the medial temporal subsystem (including the entorhinal cortex and hippocampus) of the DMN (Greicius et al., 2004; Mondragón et al., 2019). fMRI studies have demonstrated dysfunctions in the DMN to be closely correlated with self-reported episodic memory deficits in persons with AD (Zhang et al., 2022). Hojjati et al. (2021) conducted a study with a data set of 303 participants which provided evidence for a double insult hypothesis, emphasizing that it is the interaction of Aβ and tau, rather than either alone, within the DMN that plays a pivotal role in AD pathophysiology. The DMN therefore appears to be quite vulnerable to amyloid and tau accumulation, while the SN is not equally influenced by amyloid pathology (Sarli et al., 2021).

The salience network, instead, tends to demonstrate increased functional connectivity, and may relate to affective changes in AD that result in heightened attention to emotionally salient stimuli (Fredericks et al., 2018) and heightened emotional contagion (Sturm et al., 2013). This is consistent with the classic theory of network dysfunction in AD; specifically, reduced connectivity in the DMN with increased activation of the salience network to preserve cognitive integrity (Liu et al., 2018; Li et al., 2020). To explore the differences in interhemispheric functional connectivity in persons with AD vs. amnestic mild cognitive impairment (aMCI), Liao et al. (2018) utilized VMHC and found significant inhibition of the DMN and excitation of the SN and ECN. Sarli et al. (2021) examined the rs-fc fMRIs of 148 participants who had AD, mild cognitive impairment (MCI), and healthy controls. They found that in AD patients, the strength of the DMN is correlated with SN volumes, consistent with the anticorrelated nature of the DMN-SN axis (Sarli et al., 2021). Similarly, Li et al. (2019) found increased inter-network connectivity between the SN and DMN or CEN in patients with MCI and AD. Furthermore, Chand et al. (2017) found that in patients with mild cognitive impairment, the triple network switched from a SN-modulated model to a CEN-modulated model. The decreased connectivity in the DMN along with increased connectivity, perfusion, and gray matter volume in the salience network, correlate with the cognitive deficits and heightened emotions and social contagion seen in AD (Fredericks et al., 2018).

## The salience network and social-emotional communication in AD

The salience network, consisting of primarily the left anterior insula, right anterior insula, and combined left and right anterior cingulate cortex (Chand et al., 2022), also includes nodes in the amygdala, hypothalamus, ventral striatum, thalamus, and brainstem nuclei (Seeley, 2019). The SN facilitates control of activities in other brain regions and cognitive processes; reorientation of attention; switching between cognitive processes; conflict monitoring; and regulation of difficult tasks (Chand et al., 2022). Behaviorally, the SN is involved in emotion processing, autonomic regulation, interoceptive awareness, and perception of pain (Voss et al., 2013). Task-based fMRI studies have further demonstrated this primary hub to be involved in “thirst, hunger, pain, bladder distention, embarrassment” and possibly amusement, compassion, tenderness, and humor (Seeley, 2019; p. 99878). It is considered a task-positive network crucial to the identification of relevant stimuli that guides behavior, responds to internal and external stimuli, and important for attention and interoception (Song et al., 2021).

The SN can be further divided in the dorsal SN, implicated in attention switching between cognitive sources, and the ventral SN, crucial for affections and emotions (Chand et al., 2017). As such, the SN is critical for socially adaptive behavior (Fredericks et al., 2018). Despite compromised memory and executive functioning, persons with AD often experience a rich emotional life. Socioemotional sensitivity is a behavioral marker of SN function in both healthy and neurologically impaired individuals (Toller et al., 2018). Moreover, theory of mind, part of social cognition that allows one to understand other people's mental states, is related to the SN (Rijpma et al., 2021). In fact, Rijpma et al. (2021) found that the structural integrity of the cinguloinsular cortex of the SN was pivotal to theory of mind and attending to social cues, which was preserved in the 31 patients with AD in their study (Rijpma et al., 2021). While studies are inconsistent, their results further confirmed that “while AD can affect memory manipulation and executive function, which are important for theory of mind, AD typically spares SN-related functions” (Rijpma et al., 2021; p. 14).

In a similar vein, Koerkamp et al. (2012) conducted a review of the literature and found that persons with AD display a compensatory mechanism recruiting greater amygdala functioning, even when amygdala atrophy is present. They concluded that the emotional enhancement effect could be preserved, and the amygdala can serve to modulate hippocampus-dependent memory in persons with AD (Koerkamp et al., 2012). Emotions, therefore, can serve as a bolster for preserved memory and social emotional communication. Noteworthy observations of persons with AD include their ability to retain emotional traces of films, maintain mutual gaze with spouses, adopt the emotions of those around them, and when negative, can manifest the behavioral and psychological symptoms of dementia (BPSD) (Fredericks et al., 2018). Furthermore, the emotional manifestations of BPSD may, in fact, be social emotional attempts to communicate unmet needs when language functions fail (Warren, 2022). Fredericks et al. (2018) found that that in amyloid positive participants, salience network hyperconnectivity could result in heightened visceral and perceptual sensitivity to salient social cues, leading to increased autonomic arousal and emotional reactivity.

## Network changes as a compensatory mechanism

In the context of neurodegenerative disorders such as AD, a theoretical framework embodies the observation that “neurodegenerating networks upregulate connectivity as partially effective compensation” (Cope et al., 2022; p. 6). In this way, brain network compensation can also translate to psychological and behavioral compensatory responses. Persons with dementia may lose some language and cognitive faculties, but the ability to express emotion is typically preserved, and can serve as a potent indicator of individual needs and feelings (Lee et al., 2019). A profound message of personhood can be translated by this sparing of the SN to indicate that persons with dementia lead rich emotional lives. Understanding that persons with dementia retain emotional traces and implicit memory despite compromised recall ability, may aid in dispelling the stigma experienced by persons with dementia. Many formal and informal caregivers receive inadequate training regarding pathology and communication skills (Eggenberger et al., 2013). The upregulation of the salience network and associated heightened affective state signifies an opportunity for providers to educate caregivers, which may create avenues to facilitate communication, psychological well-being, and improve quality of life care for persons with dementia. Moreover, these heightened affective states, if reflective of brain disruption, may also serve as early indicators of neurodegeneration. A literature review by Johnston and Narayanasamy (2016) found that psychosocial interventions hold the potential to enhance personhood by acknowledging the person behind the patient, facilitating meaningful engagement, and offering aspects of legacy. Thus, psychosocial interventions that utilize increased salience by emphasizing social emotional understanding and engagement may foster a positive subjective experience in the caregiver-patient dyad. Taken together, the understanding of salience network connectivity may offer a lens of adaptivity in persons with dementia, rather than one restricted to pathology for medical professionals. Shifting to this perspective could inform caregivers about the psychological behaviors, communication attempts, and possible psychosocial interventions to implement for persons with dementia.

## Discussion

This brief review has several limitations. While much research has recently evolved to identify patterns of network aberrations in persons with dementia, the underlying pathological mechanisms of the triple-network in MCI and AD are still under exploration. Additionally, this narrative review is an attempt to offer insight into the relationship between brain network pathology and the subjective experience of persons with dementia, the subjectivity of which is a limitation in and of itself. Moreover, this is by no means a comprehensive review of the literature, but rather a review of current evidence through a person-centered lens. Numerous reviews offer important neurobiological underpinnings of network disruption in MCI and AD, and as such it was an aim of this paper to provide an additional layer of psychological and humanistic perspectives to contribute to care practice insight. Other important brain networks, such as the dorsal attention network and sensorimotor networks were beyond the scope of this paper and therefore not discussed. Future work may prove advantageous to include a more comprehensive review of brain networks beyond the tri-network model. Utilizing this intersection of neuroscience, psychology, clinical practice, and public education may further augment person-centered care for persons with dementia.

AD can be an overwhelming challenge to persons experiencing the disease, as well as the caregivers witnessing their decline. Much emphasis has been placed on the faculties that persons with dementia lose, rather than the cognitive and social strengths they retain (Sabat, 2019). Indeed, a lack of understanding of the person with the disease poses numerous challenges to self and personhood (Sabat, 2019), but also understanding how the disease affects a person's subjective experience may improve empathy and communication. However, understanding the neurobiological basis for behavior, along with their language of communication, can lend an insight into the subjective experience of persons with dementia that can be relayed to caregivers, loved ones, and various medical professionals. This language undoubtedly includes emotions in persons with dementia. Anecdotally, the sentiment of “it doesn't matter because they won't remember anyway” is a frequent misconception, and seems especially pertinent to the importance of educating the public regarding the emotional impact of behaviors and interactions. Implications for persons with MCI are such that signs of SN compensation may appear early in the disease process, which, in the form of heightened emotional responses may be a form of communication and coping due to the deterioration of other brain areas and networks (i.e., hippocampus and DMN). The ability to understand the communication efforts and coping behaviors used by persons with dementia, along with their neurobiological basis, may serve as educational opportunities to facilitate positive interactions and increase quality of life for both caregivers and persons with dementia.

## Author contributions

The author confirms being the sole contributor of this work and has approved it for publication.

## Conflict of interest

The author declares that the research was conducted in the absence of any commercial or financial relationships that could be construed as a potential conflict of interest.

## Publisher's note

All claims expressed in this article are solely those of the authors and do not necessarily represent those of their affiliated organizations, or those of the publisher, the editors and the reviewers. Any product that may be evaluated in this article, or claim that may be made by its manufacturer, is not guaranteed or endorsed by the publisher.
